# Supplementary material for: Extensive recoding of dengue virus type 2 specifically reduces replication in primate cells without gain-of-function in Aedes aegypti mosquitoes
Source: PLoS One. 2018 Sep 7;13(9):e0198303. doi: 10.1371/journal.pone.0198303 (PMC6128446; doi:10.1371/journal.pone.0198303)
Supplement: S1 Fig — At 2 days post infection, infected cells were fixed and stained using 4G2 anti-E or anti-NS3 primary antibodies as well as Alexa 488 (E) or fluorescein isothiocyanate (NS3) conjugated secondary antibodies. (PDF) [file pone.0198303.s001.pdf]

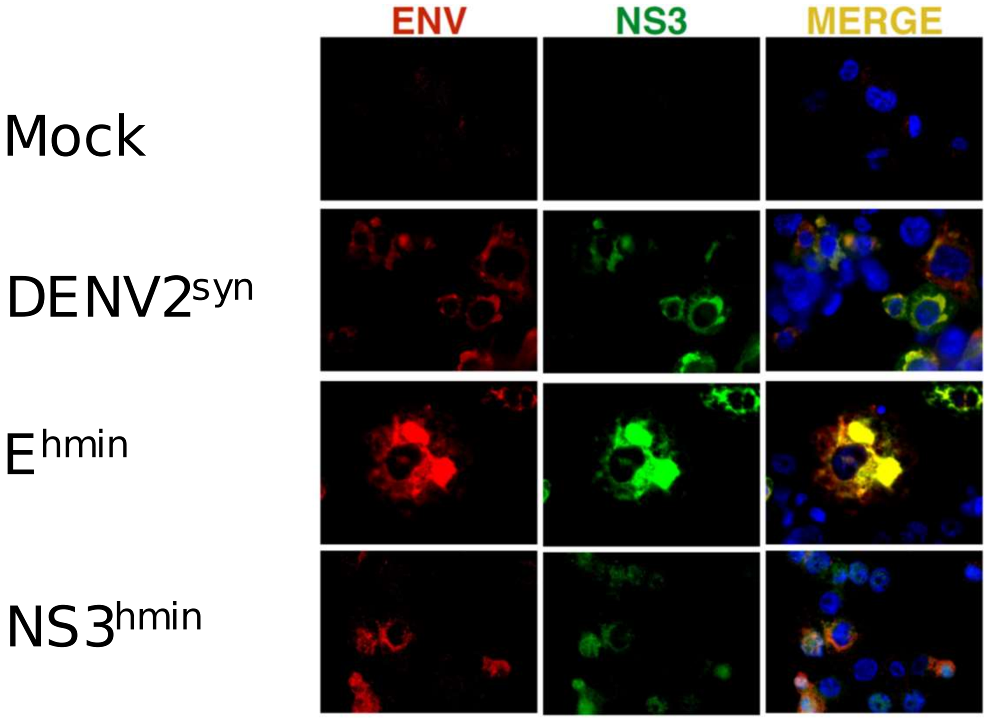

Figure S1. Deconvolution microscopy of THP-1 cells infected with D2-Syn, E hMin , and NS3 hMin or mock infected at an MOI of 5.0. At 2 days post infection, infected cells were fixed and stained using 4G2 anti-E or anti-NS3 primary antibodies as well as FITC (NS3) or Alexa 488 (E) conjugated secondary antibodies.
